# Design, development, and testing of a voice-text mobile health application to support Tuberculosis medication adherence in Uganda

**DOI:** 10.1371/journal.pone.0274112

**Published:** 2022-09-09

**Authors:** Kenneth Kidonge Katende, Mercy R. Amiyo, Sarah Nabukeera, Ian Mugisa, Patrick Kaggwa, Stellah Namatovu, Simon Peter Atwiine, Simon Kasasa

**Affiliations:** 1 AIDS Clinical Trials Group, Data Department, Joint Clinical Research Centre, Kampala, Uganda; 2 Department of Information Systems, School of Computing and Informatics Technology, College of Computing and Information Sciences, Makerere University, Kampala, Uganda; 3 Department of Epidemiology and Biostatistics, School of Public Health, College of Health Sciences, Makerere University, Kampala, Uganda; 4 Information Technology, Uganda Virus Research Institute, Entebbe, Uganda; National University of Singapore, SINGAPORE

## Abstract

**Background:**

Tuberculosis (TB) continues to persist with a high disease burden globally. Non-adherence to treatment remains a major problem to TB control. In Uganda, one in every four TB patients does not adhere to their TB medication. The purpose of this study was to design, develop and assess implementation of a voice-text-based mobile application to support TB patients’ adherence to medication.

**Methods:**

Design science research methodology (DSRM) was utilized to develop a voice-text-based mobile health application. Agile software methodology was used to achieve steps of DSRM that are; design and development. Focus group discussions (FGDs) and Key informant interviews (KIIs) were conducted and data analysed using thematic content analysis.

**Results:**

During problem identification, Stigma, transport costs, being asymptomatic, drug side effects, lack of family support were identified as challenges affecting adherence. Technologies identified and used for the development of the voice-text application included; extensible mark-up language (XML) File, Apache server, Ubuntu Server, Hypertext Pre-processor, and jQuery. In the pilot study, 27 voice messages were broadcasted, 85.2% were delivered, 103 text messages were sent and 92.2% were delivered to the intended recipients.

**Conclusions:**

Voice-text message mobile health application can be used to reach a wider patient population and it has the capability of addressing some of the challenges affecting TB medication adherence.

## Introduction

Tuberculosis (TB) is a major disease burden globally. About 10 million incident cases were reported in 2019 and a quarter of the cases were found in the African region [1]. In Uganda, TB incidence stands at 200 cases per 100,000 population [1]. One of the major problems affecting TB control is non-adherence to treatment which leads to the occurrence of multidrug-resistance TB [2]. One in four TB patients in Uganda are non-adherent to their treatment [3], and this is one of the reasons for the slow reduction in transmission of TB. Digital adherence technologies such as pillboxes, video directly observed therapy (VDOT), text and voice messages, and ingestible sensors have been fronted as interventions to support patients’ adherence to medication [4].

Directly observed therapy (DOT) is the standard intervention that was established by the World Health Organization (WHO) to ensure treatment adherence, and Uganda attained full district coverage of DOT in 1995 [5]. However, the proper implementation of DOT has proved difficult to achieve especially in Sub-Saharan Africa [6]. The major challenge affecting DOT in Sub-Saharan Africa is the limited healthcare worker force, as the in-person observation makes DOT a labour-intensive and time-consuming activity [7–9]. In Uganda, only 16% of the health centres could properly implement DOT due to the limited number of health workers [10]. The other challenge is stigma in picking treatment supporters as patients feel these may disclose their TB status to other community members [11, 12].

There are other challenges affecting TB medication adherence such as the traveling long distances to healthcare facilities resulting in costly transportation fares which discourages patients from returning for drug refills hence missing their medication [9, 13, 14]. There is lack of social support from family and community for TB patients, as well as and the impact of drug side effects such as nausea, vomiting, body weakness, which have led to patients abandoning taking medications [15–17]. Lack of adequate knowledge about TB has contributed to non-adherence to TB medication[15–18], as some confuse TB with human immunodeficiency virus (HIV) while others think the disease is hereditary [17]. Some of the patients simply forget to swallow their medication [15] due to busy schedules [18], while for are unable due to excess alcohol consumption [19]. Additionally, after the intensive phase (2 months of isoniazid (INH), rifampin (RIF), pyrazinamide (PZA), and ethambutol (EMB)), patients tend to feel that they have been cured due to the disappearance of most of the symptoms [17, 19], and as such begin to miss taking their TB medications. These are some of the factors contributing to the 26% non-adherence to TB medication in Uganda [3].

WHO developed a handbook to highlight and encourage the development of digital adherence technologies [20], to support patients taking their medication in accordance with the prescription given to them. Globally, there is an increase in the use of mobile technology to provide health services and Sub-Saharan Africa has experienced a rise in accessing mobile technologies that have led to the creation of significant opportunities for the development of cost-effective mobile health (mHealth) interventions [21]. Studies have reported on the feasibility, acceptability, and validity of the different kinds of mHealth intervention in rural Africa [22]. Mobile Health tools that use text messages have been developed to support patients to adhere to medication mainly for people living with HIV [22].

In Uganda, digital health interventions have been used before; to link pregnant women to the nearest primary health care facility for childbirth [23], remind women of antenatal care (ANC) appointments, support antiretroviral therapy (ART) adherence, and provide patients with information about healthcare centres that provide HIV-related services [24]. SMS messages have been used in health promotional activities like creating awareness of HIV, family planning services [25].Village health workers have used smartphones to register pregnant women and also track them by using SMS [23] to improve maternal health services in Uganda. Studies have also shown that Video DOT is feasible, acceptable for supporting TB treatment adherence, and can increase adherence levels to over 90% [26]. Pillboxes have demonstrated effectiveness in improving medication adherence among TB patients [27] and were found acceptable, satisfactory, easy to use, and with very low likelihood of missing medication [28, 29].

Community-based peer health workers (PHWs) have used mobile phones either to call or text HIV patient-specific clinical information to healthcare centers that provide HIV-related services to improve the overall care of HIV patients [24]. Generally, mobile telephone interventions i.e. texts help in increasing rates of treatment success, medication adherence, and 2-month sputum conversion when used as tracers [30]. Thus, the voice-text application could help to overcome some of the challenges affecting DOT and medication adherence in general. However, there is a dearth of information on the design and development of the voice-text-based mobile health adherence support application, in resource-limited settings. This study aimed to develop, design, and asses the implementation of a voice-text-based mobile health application, to support adherence to medication among TB patients in Uganda.

## Materials and methods

### Study setting

Uganda’s population stands at 42.8 million [31] while its mobile phone subscription stands at 25 million [32], making it feasible to develop and implement the voice-text mobile health applications which weren’t previously reported as combined tools in our settings to support medication adherence. The research was done at the Joint Clinical Research Centre (JCRC), which has various treatment clinics including; the TB clinic that attends to both HIV-negative and positive TB diagnosed patients was restricted. The organization provides ART drugs and offers treatment of opportunistic infections to over 200,000 patients around the Greater Kampala Metropolitan region (Kampala City, Mukono, Wakiso, and Mpigi Districts). Adherence support is provided through trained community health volunteers and family members or friends to the patients.

### Study methodology

Design science research methodology (DSRM) was utilized to develop a mobile health voice-text-based application following six consecutive steps, namely: problem identification, objectives of a solution, design and development, demonstration, evaluation, and communication [33]. Design science is a problem-solving process. It requires the creation of an innovative, decisive artifact for a specified problem. The artifact must produce a utility for the specified problem thus requiring thorough evaluation of the artefact. The processes involved in constructing innovative IT artifacts, enables researchers to understand the problem addressed by the artifact [34]. DSRM was suitable for this kind of research because non-adherence to TB medication is a real-world problem and designing as well as developing a voice-text mobile application can help tackle TB medication non-adherence. The voice-text mobile health application was operationalized in a prototype and implemented at the Joint Clinical Research Centre as a case study. Four focus group discussions (FGDs) and eight key informant interviews (KIIs) were conducted during the different steps; problem identification, requirements identification of the voice-text and mobile health application and the usefulness of the application.

#### Text messages decision

During the designing process, a series of messages were drafted through discussions amongst the design team (researchers, telecom expert, software developer, TB focal person, counsellor, and Clinician) using data from KII, FDGs and literature, as shown in Table 1 below. These were used during the testing of the TB medication adherence mHealth Tool.

**Table 1:** Voice-text messages for the different categories.

Category	Messages
**Medication**	Dear friend, please take the pills as instructed by the healthcare worker otherwise the disease will become stronger and resistant.
	Hello dear, please remember to take your TB drugs today. Don’t share your medication with anyone. Thank you
	This is a simple reminder to you to continue taking your medications as this protects you from getting dangerous TB.
	Remember to take your drugs today as TB cures only if you take all drugs daily without missing them. Keep on taking them every day without stopping!
	TB is curable once you take the drugs daily without missing. Please remember to take your dose today.
**Appointment**	Hello Dear! Your health is above everything. We are waiting for you at the Health Centre on **XX-XXX-XXXX**!
	Good health gives joy. We are anxiously waiting for you. Please, remember to come to the health Center on **XX-XXX-XXXX**.
	Hello dear, TB drugs are free, and TB cures. We shall be happy to see you as we are waiting to see you for your appointment today **XX-XXX-XXXX**.
	Hello! Remember to come and collect your medications to enable the cure of TB today **XX-XXX-XXXX**.
**Education**	Dear friend! You must maintain a healthy lifestyle. Do not drink alcohol, do not smoke, get enough rest, drink lots of water or juice.
	Always when coughing, do it in the elbow and when sneezing, cover your mouth with a handkerchief to avoid cross-infection.
	Friend, TB is not a family disease and you cannot get it through handshaking. Oh, remember this, Herbalists do not cure TB disease.
**Motivation**	Congratulations on completing the intensive. Don’t give up! You may feel better but you are NOT CURED. Keep taking your medications, dear! We know you can do it!
	Great job, dear! You are almost done! You have completed **XX** weeks of treatment. Keep the good spirit of coming to pick your drugs!
	You are now in the maintenance phase and the number of pills has reduced. Keep it up dear!

#### Problem identification

This DSRM step defines the specific research problem to rationalize the worth of the anticipated solution and develop an artifact to help solve the problem [33]. Three FGDs and 8 KIIs were conducted among TB patients and healthcare workers respectively, to understand challenges affecting TB medication adherence.

*Requirements gathering/elicitation*. Designing mobile health applications begins with understanding the requirements of the user (TB patients and partly healthcare workers). All the KIIs and three FGDs centred on the four categories of messages that is; medication reminders, appointments, education, and motivation, focusing on content, timing, frequency, and order for each of the categories.

#### Design and development

Under this step, the actual development of a solution in the form of the artifact was done. Agile software methodology was used in the development of the application [35] to enable software developers to consider the input of the different stakeholders that is; doctors, nurses, counsellors, the information technology expert, the telecommunication expert and TB patients. During the brainstorming sessions the development team that was composed of: TB focal-person, clinician, a software developer, information technology expert, telecommunication expert and led by the first author discussed the different technologies required for the development of the voice-text based mHealth application. During the discussions, other usability requirements were identified and the process continued iteratively throughout the designing and development of the system.

#### Demonstration and testing

A demonstration and training on the voice-text mHealth application were performed for the TB clinic healthcare workers before the application was pilot tested. Voice and text messages were sent for one month.

### Selection of study participants and data collection

KIIs were conducted amongst the healthcare workers aged 18 years and above working in the TB clinic; to get their perspectives on the challenges affecting TB medication, content, frequency, timing, and order of the voice-text messages. The healthcare workers were purposively sampled based on their knowledge, expertise and experience, by a trained research assistant during the TB clinic working days (Tuesdays and Thursdays). KIIs were conducted in English in a private setting after each participant consenting and these lasted between 28 to 35 minutes. All the KIIs were audio-recorded and transcribed verbatim.

FGDs were conducted amongst TB patients in a quiet private setting; to understand the reasons for non-adherence, opinions on the kind of content, frequency, timing, and order of text-voice messages. Patients on the intensive phase of TB medication aged 18 years and above were conveniently sampled by a trained research assistant as they came into the clinic for their drug refills and consented to participate in the study. Each FGD consisted of 7–8 participants, a moderator, a note-taker, a timekeeper, and lasted between 60 and 70 minutes. All the FGDs were done in a local language (Luganda), audio-recorded, transcribed verbatim, and later translated into English.

The first author supervised all the data collection activities and constantly checked the transcripts for quality, detail, and grammatical errors. The authors listened to a sample of audios at the start of data collection to check for completeness and quality of information. The first author further reviewed transcripts for accuracy and all inconsistencies were corrected accordingly, on time.

### Data analysis

After each FGD or KII, summary notes of the conversation were written highlighting key themes (emerging and apriori), standardized summary worksheet. To ensure quality, the notes were proof-read notes by a second person on the team. Data were later manually analysed using thematic content analysis employing a deductive and inductive approach. Some of the apriori themes were: challenges to TB medication, content, frequency, timing, and order of messages.

### Ethical considerations

This study was approved by two accredited Research Ethics Committees (RECs); the Higher Degrees Research and Ethics Committee at the Makerere School of Public Health, and the Joint Clinical Research Centre Regulatory Ethics Committee, Kampala, Uganda. Participants provided written informed consent in either English or Luganda according to the language they understood.

## Results

Out of the 22 participants enrolled to participate in the FDGs, 63% were male, and 45% were aged between 40 to 49 years, while 13% had no formal education as shown in Table 2. Eight KIIs were conducted among the healthcare workers; across the characteristics of Sex; Female or Male, Age; 20–29, 30–39, 40–49 and 50–59, and Profession; Nurses, Counsellors, Doctors, Health visitors, and Pharmacists. We don’t provide the number of participants per characteristic category for the key informant interviews to avoid deductive disclosure; due to the small number of participants that is, one participant, in various categories for some characteristics.

**Table 2:** FGDs participants characteristics.

Age	FGD Participants N = 22(%)
20–29	4 (18.2)
30–39	6 (27.3)
40–49	10 (45.5)
50–59	2 (9.1)
**Sex**	
Female	8 (36.4)
Male	14 (63.6)
**Education Level**	
None	3 (13.6)
Primary	12 (54.6)
Secondary	7 (31.8)

### Problem identification

The following challenges were identified by TB patients and the healthcare providers.

*Stigma*: All the key informant interviewees (8/8) mentioned stigma but also 40% (9/22) of the FGD participants mentioned stigma as a challenge which was explained as hiding, avoiding, fear, not being seen while taking TB medications discrimination, and separation of utensils. For example, one FGD participant said;

*“Another was saying that we live so many at home*, *and you are given so many drugs which are also big*, *you don’t have anywhere to keep them and hide them*, *meaning they still have the stigma*. *Also*, *the doctor said we should avoid the kids*, *neighbours so that you don’t spread to them and you decide to hide from them thus some tending to abandon the drugs*.” (Participant–Focus Group)

*Transportation costs*: Eleven out of 22 FGD participants and more than half (5/8) of the key informant interviewees mentioned transportation costs to visit the health centres as a challenge. This was highlighted as lack of transport funds, failure to get money, transport means problem and much-hiked transport fees since majority stay far away from the health centres as one FGD participant explained;

“*Another reason that I think about is the distance from where we stay and we might not have transport to bring us to the place where we pick our drugs from and we fail to pick the drugs due to lack of transport funds we fail to come to pick our TB drugs*.*”* (Participant–Focus Group)

*Feeling better (Asymptomatic)*: Over 50% (11/22) of the FGD participants acknowledged the issue of the ‘patients feeling cured or better’ after being on the drugs for a certain duration. This was explained as; gaining strength and weight, feeling better, gaining appetite, symptoms going away, and feeling okay.

*‘Some other patient told me that he took the drugs and felt better*, *and asked why should I continue with the drugs*? *And this was after taking for two weeks and he had gained some strength*, *appetite and decided to see through the dose and said he is done*.’ (Participant–Focus Group)

*Drug side effects*: More than 50% of FGD participants (12/22) and 62% of the KI interviewees (5/8) raised the concern of patients missing taking their drugs due to side effects from the drugs. Side effects such as numbness, joint pains, and blacking out, nausea were highlighted and for example, one participant said;

“*These drugs bring side effects which are problematic like joint pains*, *numbness in the legs*. *If someone is the breadwinner at home*, *gets side effects*, *and cannot allow him to go to work*, *he will then decide to abandon the drugs so has to go back to work to earn something to be able to feed the family*.*’* (Participant–Focus Group)

*Lack of information*: Lack of general knowledge or enough information regarding TB among patients was expressed as; didn’t know how TB is spread, the mechanism of action of TB drugs, and the importance of adherence.

*‘A number of our clients are illiterate*, *remember TB tends to affect the poor societies and they tend to have lower education levels and with less knowledge comes a challenge of understanding why I should be on this medication every day*.*’ (*Doctor TB Clinic KII006)

*Lack of family/community support*: Four out of the 8 KI interviewees and 7 out of 22 FGD participants pointed out lack of social support as one of the barriers to TB treatment adherence. This entailed a lack of care from the community, peers, or family, and that many times there was no one to remind the patient to swallow their drugs as explained by one of the KI interviewees that;

‘*lack of family support or support from peers*. *Yeah*, *because someone may not be living with family but when they have peers around if a person does not get support*, *I think it affects adherence in a way that you are not motivated to move on and continue swallowing your medicine*.’ (Nurse KII001)

### Design and development

#### Content of voice-text messages

In the appointment category, over 50% (12/22) of the FGD participants and 3 out of 8 KI interviewees who answered this question emphasized the date of return to the clinic as the most important aspect. This was highlighted as the date of return to the clinic, remind of return, and don’t forget to come tomorrow and time to come to the health facility.

*“I am sending a message to my patient*, *it should be brief and carrying the correct information like “my wonderful patient am reminding you on such a date you have a visit in our clinic*. *Kindly asking you to keep time and we shall also attend to you very first*.*”* (Participant–Focus Group)

The main emphasis in the medication category was to remind patients and encourage them to take their medication promptly. This was stated by all (8/8) the KI interviewees and 13 out of 22 of the FGD participants as the time to take medication, remind and encourage to take medication.

*“If am texting as the healthcare provider*, *I will tell him that Mr*. *X am reminding you*, *did you take your medication at the rightful time*? *The drugs are your life*! *If you miss your drugs forget about your life but I encourage you to take medications at the right time and you will be cured*.*”* (Participant–Focus Group)

Fifteen out of 22 FGD participants and 6 out of 8 KI interviewees stressed the importance of what is TB, prevention of TB, how TB is cured, the signs and symptoms of TB, and the importance of adherence as the vital content for the education category. For example, one participant said;

*“If I don’t know anything about TB and it is my first time and he is educating me*, *I do expect him to tell me*, *what is TB*? *What causes TB*? *How is TB cured*? *How do we prevent TB from spreading*? *After telling me that*, *maybe the time is telling me all this*, *I might have the symptoms and should inform me about the signs and symptoms of TB*.*”* (Participant–Focus Group)

The motivational aspect was appreciated and the primary emphasis was mainly on the duration one is remaining and has spent taking the drugs.

*“First thank him for having taken the drugs and inform him that the good thing about TB is if you adhere to your drugs and you don’t miss or reduce the dose*, *Tb cures*. *If you adhere well to your drugs and finish the 6months they will repeat to test TB to see if has cured or not cured*. *If it has not been cured*, *it means you didn’t take properly the drugs*, *or you have been skipping some days but the good thing about TB is that it cures*.*”* (Participant–Focus Group)

#### Frequency

The most suggested number appointment messages were three and it should be before the return date as mentioned by one of the Key Informants;

*“I think appointment should be maybe like 3 days before their next appointment then you will be reminding them that two days from now is your appointment when it’s one day*, *you tell them that tomorrow is your appointment*.*”* (Nurse TB Clinic KII002)

Less than a quarter of the participants suggested that medication reminders be sent; once a day, daily, every day, three a day, and twice a day.

*“Is it possible to know the time when this particular patient is going to take the medication so that it can act as a prompter*, *Tune the person not to take the medicine late*? *They could be sent daily*. *If the person is not just stubborn*, *he will take the medication*… *if forgetting is the reason*… *then it would work*.*”* (Counsellor KII005)

Eight out of 22 of the FGD participants suggested that educational messages be sent either before or after a patient visiting the healthcare center but not daily.

*“It can be every time they are coming to the appointment or after they have gone after the appointment*, *you send a message…these education messages are always good*.*”* (Pharmacist KII007)

The frequency for motivational messages was preferred; after a visit and before an appointment.

“*These messages will work best before the cycle ends or at the start of the cycle and the patient is about to return or has just been at the clinic for a refill*. *One or two such kinds of messages will be enough*.*”* (Nurse/Counsellor KII004)

#### Timing

The preferred time for an appointment, 10 out of the 22 FGD participants and 3 out of 8 KI interviewees were of the view that these messages should be sent in the evening and morning time on the day of the appointment.

*“Like someone should wake up*, *I believe most people wake up around six*. *So*, *this message should be around six*, *yes if it’s not possible let it be like late in the evening*, *but not during when people have their other things to do*.*”* (Pharmacist KII007)

The medication reminder should be sent at the time of medication or one hour before the patient takes his or her medication since this can act as a prompter, treatment supporter for the patient to know and stick to the time of medication according to 12 out of 22 FGD participants and 4 out of the 8 KI interviewees.

*“I think because these patients have particular times they prefer because I always ask them they should choose a time that is convenient by themselves*, *so once you know that means it will be taxing for you people sending the messages*, *they will not be sent at the same time because of the different patients timing so I would think maybe*, *few hours or some few minutes to the person-time of taking the drugs*, *the message is sent*, *so we should*. *be like a reminder*” (Nurse TB Clinic KII003)

Eleven out of 22 FGD participants suggested that educational messages should be sent in the evening or morning due to a lot of distractions during the day. This was also echoed by 3 out of the 8 KI interviewees.

*“It’s either the morning or the evening because during the day we have lots of distractions*, *but then most of these TB treatments are taken in the morning*, *so if*, *in my opinion*, *it would be morning hours*, *especially if they take it in the morning*.*”* (Doctor TB Clinic KII006)

A third (7/22) of the FGD participants and 2 out of 8 KI interviewees suggested that motivational messages be sent to patients in the morning before medication, evening, before sleeping like 8 pm because in the day people are so busy.

*“Most patients don’t usually take their medications very in the morning*, *they take their drugs around after eating something at 8 am*, *9 am and 10 am but if you motivate him with a message around early morning*.*”* (Participant–Focus Group)

#### Order of categories

Eleven out of 22 FGD participants and 3 out of 8 KI interviewees who answered this question acknowledge that the order in which to deliver these messages did not matter and that wouldn’t affect the messages, as long as they read it, any category you start with is good enough, moreover, they have different meanings.

*“Any category that you start with is good enough no need for any particular order*. *Any one of us who is here*, *everyone one wants to be alive thus everyone has to caution himself that on this day I have to go back and pick my drugs because once you don’t pick your drugs*, *your situation will deteriorate because no one is going to push you to go pick your drugs*.*”* (Participant–Focus Group)

### Development of mHealth system

After the brainstorming sessions the development team came up with the following technologies to develop the voice-text based mHealth application;

*XML File*: The voice messages were stored in an XML file on the Linux server. TwiML also an XML file transported the voice message from the XML file on the Linux server and instructed Twilio to broadcast information as an outgoing call.

*Twilio*: The development team looked at the different open-source platforms for text messaging systems but all had one limitation, not being able to broadcast voice. The team decided to use an aggregator that can provide the service of voice and text. Twilio was selected as it is flexible, relatively cheaper than other available aggregators on the market and it has been used in Clinical Trials database systems such as Research Electronic Data Capture (REDCap).

*Apache HTTP Server (Web Server)*: This open-source cross-platform web server is well-optimized and capable of handling a large amount of traffic. It hosted the TB medication adherence mHealth application since it’s web-based and responsible for establishing a connection between a server and the browsers of the website.

*Ubuntu Server*: An open-source server operating system that was used to generate automated voice and text messages courtesy of a cron job. A cron job is a Linux utility that schedules a script on a server to run automatically at a specified time and date. The Ubuntu server was also selected because it provides lesser threats over the internet in terms of security, ease of access to tools such as PHP and *MySQL*: It hosted the XML file, Apache HTTP Server, MySQL database server, and the mHealth Application.

*PHP (Hypertext Pre-processor);* was used to create the web user interfaces and the functionality of the TB medication adherence mHealth application.

*jQuery;* was used interchangeably with PHP to create web user interfaces and for the functionality of the TB medication adherence mHealth application. It was used for specific JavaScript function effects.

#### System functionality

**Fig 1:**
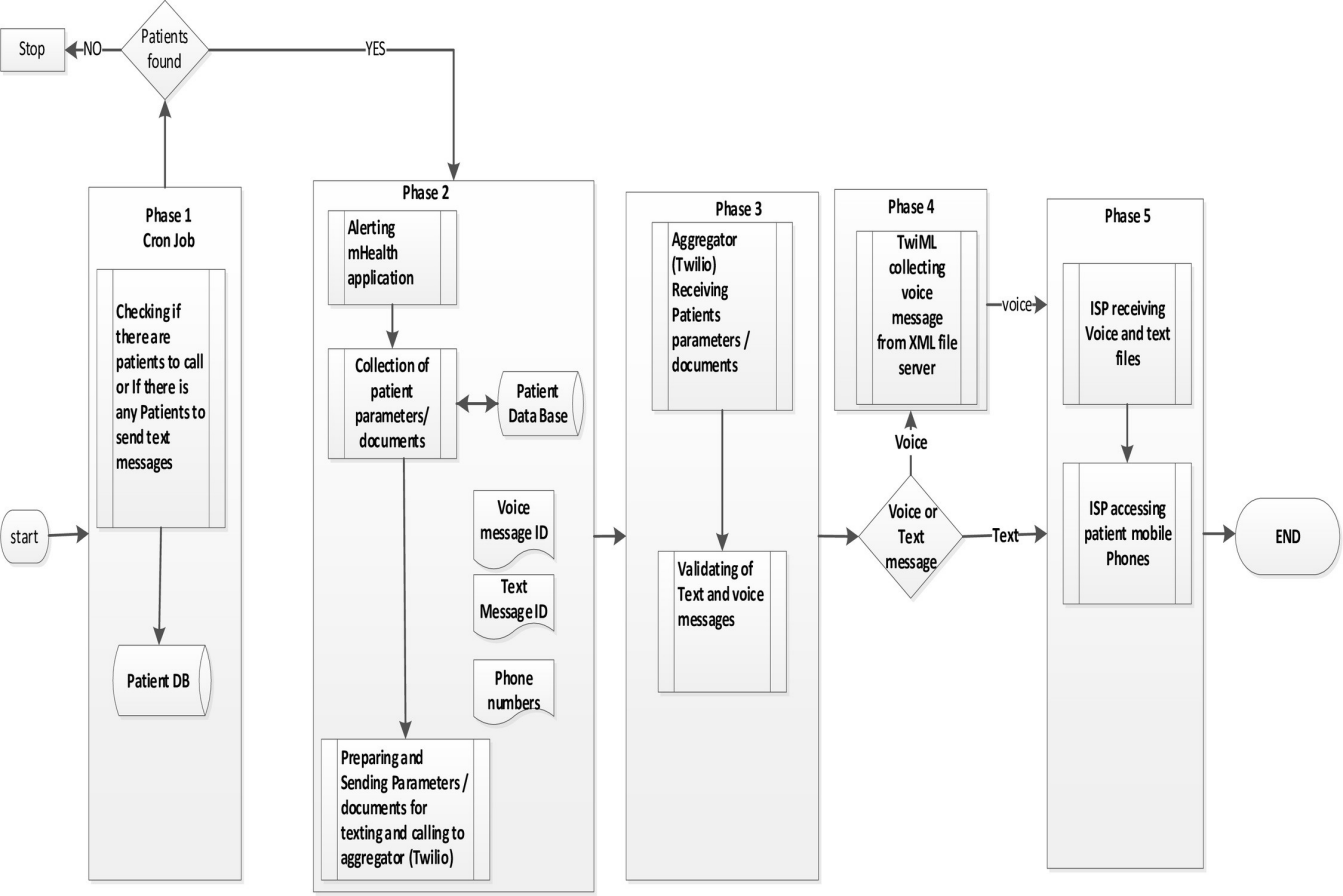
Process flow diagram of sending a text message and broadcasting voice messages.

A cron job was created at the Linux server and ran every minute to check the patients’ database if there was anyone due for either a voice or text message. If a patient was found, an alert was sent to the mHealth application which collected the parameters of the participant from the patients’ database such as text or voice message ID and patient’s mobile number which were then sent to the aggregator (Twilio). The parameters were then validated by Twilio, if it was a text message then it was sent to the patient’s mobile via the telecom provider, and for a voice, Twilio sent the voice ID to the XML file located on the Linux server. The TwiML functionality collected the voice message content from the XML file on the Linux server and sent it to the patient’s mobile via the telecom provider. The patient’s telecom provider would then deliver either the text to the patient’s mobile phone or broadcast the voice message. The process flow of information is accommodated within the architectural diagrammatic presentation in Fig 1 below.

#### System implementation

A system administrator registered a counsellor and a nurse by assigning them a username and password to register patients on the voice-text mHealth application. Upon registering the patients, the system created a schedule of voice and text messages to be sent for the entire medication duration (six months).

### Demonstration and testing

The demonstration of the voice-text message mHealth application was done at the Joint Clinic Research Centre through training the healthcare workers in the TB clinic. Nine patients on the intensive phase of TB medication who owned mobile phones were purposively sampled as they came for their drug refills and these were consented to receive voice and text messages. Six were male, five opted to receive voice/text messages in English, and 8 understood both English and Luganda.

#### Voice messages

Only education and motivational messages were sent for the voice feature. A total of 27 voice messages were broadcasted to the participants, 51.9% received by the recipient, 14.8% failed due to probably the phone had been switched off as shown in Table 3. The busy and no answer messages when considered then 85.2% of voice messages got to the designated recipients.

**Table 3:** Voice messages sent to participants.

Status	Frequency	Percentage (%)
Busy	1	3.7
Completed	14	51.9
Failed	4	14.8
No Answer	8	29.6
Totals	27	100

#### Texts messages

A total of 103 text messages were sent, 92.2% were delivered as illustrated in Table 4 below. The undelivered messages were due to an unreachable destination handset and an unknown error from the telecom provider.

**Table 4:** Text messages sent to participants.

Status	Frequency	Percentages (%)
Delivered	95	92.2
Undelivered	8	7.8
Totals	103	100

### Participants’ perception

An FGD (See: S1 Appendix) was conducted at the end of the testing period to understand the participants’ perceptions about the mHealth application. The patients were; aged 18 years or older, 2) had basic literacy, 3) on TB medication, 4) owned a cell phone with voice and text message capabilities, 5) not part of another ongoing research. Almost all (8/9) believed that the initiative of voice-text messages is a good idea, a good program that should continue and would help remind them.

*“This thing is nice because if at my first episode of TB was receiving these messages*, *maybe I would already be cured of TB*. *In the first episode*, *I took the drugs and was told that the drugs might not have worked but I think the problem was that I thought that had cured before completing the dose*.*”* (Patient–Focus Group)

More than half of the participants (5/9) explained the usefulness of the mHealth application as; tailored in a way that I like, it encouraged us, gave us strength, and what we’re being told is truthful.

*“It was useful in such a way that I was able to know more about TB because these messages were tailored in a way that I liked*. *Today you reminded me that I have TB then tomorrow that I should take my drugs*. *This was useful that when you read the text message that TB is curable*, *even if you tend not to take the drugs*, *it encourages you to ask for water to take your medication*.*”* (Patient–Focus Group)

Two-thirds of the participants (6/9) expressed that the mHealth application provided a good feeling of being cared for by their healthcare providers.

*“I felt happy*. *I felt so happy because I knew that my healthcare providers were thinking about me and care about my wellbeing*. *This motivated me to keep taking my TB drugs and care about myself because they had shown they care by sending messages and yet they are not my parents”* (Patient–Focus Group)

Participants expressed their views to improve the TB medication adherence mHealth application in terms of content and delivery time of the voice-text messages as one pointed out that the messages should be sent the exact time a patient is expected to take his or her medication.

*“So*, *if a patient tells you that I take it at 7 am or in the evening*, *you send that message exactly the time they take the medicine*.*”* (Patient–Focus Group)

However, a third (3/9) of the participant raised the issue of the voice message delivered by the mHealth application being short and brief as one participant noted below.

*“You can always call on us to give you feedback but for the voice message*, *it was short and brief whereby someone could not get the message very well*.*”* (Patient–Focus Group)

## Discussion

In this study, stigma, transport costs, lack of social support, being asymptomatic, the poor attitude exhibited by the healthcare workers, lack of information, and drug side effects were identified as challenges affecting adherence. Similarly, studies conducted in other parts of sub-Saharan Africa have shown similar challenges such as lack of knowledge, stigma, lack of social support, poor patient-provider relationship, and drug side effects affecting TB medication adherence [16–18]. This connotes the need for targeted health interventions by TB care programmers to find workable solutions to these challenges and ensure optimal adherence to TB medication.

Personalization of messages was a key component concerning message content development as the emphasis was return date to the clinic, remind and encourage, duration one has spent taking the drugs. A previous study showed that participants rejected generic messages opting for individualized messages [36]. In this study, we also established that; for the medication messages to be effective, they should be sent daily at the time of patient medication intake to act as reminders. This was in agreement with another study whereby patients preferred daily messages [37] because they act as a reminder to take medication. This study’s findings also emphasized that appointment messages be sent for three consecutive days including the appointment date before returning to the clinic. This stressed the importance of taking patients’ perspectives into account when developing systems that extend the reach of care by programmers.

During the testing phase, the voice-text message mobile health application was found to be able to deliver adherence support to more people than DOT as over 85% of the voice and text messages were delivered to the intended recipients compared to the 66% (133 patients) of DOT [3]. The voice-text message mHealth application was also able to provide a good feeling of being cared for by their healthcare providers as expressed by two-thirds (6/9) of the FGD participants thus providing social support. This means that some of the challenges that were identified like stigma, transport costs, lack of social support can be addressed by voice-text message mHealth application as shown by a study conducted in South India [38]. By using XML File, Apache HTTP Server (Web Server), PHP MySQL, jQuery, and Ubuntu as development tools, we were able to demonstrate the use of open-source software in developing low-cost mobile health applications. Application developers in limited-resource countries should look out for open-source software to minimize costs.

### Strength and limitations of the study

The technical limitation of our TB medication adherence mHealth application was failure to personalize the voice messages as the design team could not automate the insertion of the names of patients. Additionally, the costs of text and voice messages are currently higher than the local prevailing rates as the researcher failed to find a local aggregator. To achieve this, we needed to have generated the text messages that already had patient names, then integrate a text to voice application within voice-text message mobile health application; and a mobile telecom provider had to be outsourced to act as an aggregator through a memorandum of understanding with the TB program, to make this cheaper.

However, the strength of the study is that; the Joint Clinical Research Centre cares for TB patients from almost, all parts of the country, and thus, these study findings to some extent, put forward the diverse views of people on TB medication in Uganda that need further scrutiny. We used an open source software to develop the voice text mobile health application; which makes it flexible, reliable, user friendly and cost effective if used to support TB medication adherence. More evidence is required to ascertain the feasibility of its application for different populations across different settings.

## Conclusions

Voice-text message mobile health application can reach a wider patient population than DOT and it has the capability of addressing some of the challenges affecting TB medication adherence. It is therefore prudent for TB program officers to add it to the DOT to achieve the End TB Program milestone, a world free of TB with zero deaths [39].

## Supporting information

S1 Appendix Focus group discussion guide to evaluate artifact. (PDF)
