# Supplementary material for: Design, development, and testing of a voice-text mobile health application to support Tuberculosis medication adherence in Uganda
Source: PLoS One. 2022 Sep 9;17(9):e0274112. doi: 10.1371/journal.pone.0274112 (PMC9462710; doi:10.1371/journal.pone.0274112)
Supplement: S1 Appendix — (PDF) [file pone.0274112.s001.pdf]

FOCUS GROUP DISCUSSION (FGD) GUIDE FOR EVALUATING ARTIFACT:

A MOBILE HEALTH TOOL COMBINING TEXT AND VOICE TO SUPPORT TUBERCULOSIS MEDICATION ADHERENCE.

I. INTRODUCTION

Welcome and thank you for volunteering and agreeing to participate in this study. My name is KENNETH KIDONGE KATENDE, a Master of Health Informatics student at the School of Public Health, Makerere University, and together with…………………………………...as the moderator and ……………….................as the note-taker, we are the research team.

The purpose of this FGD is to find out your thoughts and views on the TB medication adherence mHealth Tool. During the discussion we shall try to understand the usability of the mHealth application. The FGD will take no more than two hours. The discussion will be taped to facilitate its recollection.

II. PRELIMINARY SECTION

Date of the FGD: …………………………………………………………………

MODERATOR: ………………………………………………………………….

NOTE-TAKER: ………………………………………………………………

FGD VENUE: …………………………………………………………………

TIME STARTED: ……………………………………………………………

TIME ENDED: ………………………………………………………………

LANGUAGE USED: …………………………………………………………

NUMBER OF PARTICIPANTS: ………………………………………………

FGD IDENTIFIER ……………………………………………………………....

III. ANONYMITY

Despite being audio recorded, this discussion will be anonymous. The tapes will be kept under double key lock, accessed only by the research team. Transcription will be done word for word and thereafter the tapes will then be destroyed. The transcribed notes of the discussion will contain no information that would allow individual subjects to be linked to any of the statements. Everyone

should try to answer and comment as accurately and truthfully as possible. All those participating in this discussion would appreciate it if you would refrain from discussing the comments of other group members outside the focus group. If there are any questions or discussions that you do not wish to answer or participate in, you do not have to do so; however please try to answer and be as involved as possible.

IV. <u>GROUND RULES</u>

1. We would like you to give us as much information as possible during this discussion.
2. There are no right or wrong answers in this discussion, so all answers are welcome.
3. We shall be audio-recording the discussion to be used for making good notes. However, whatever is said here stays here and only people who belong to the study team will access the recording and your names will not be mentioned outside this discussion.
4. When discussing please make sure you stick to the subject.
5. We shall have one person talk at a time.
6. You can tell us what you think, what other people think, what you know or what other people know about the subject.
7. You may share stories, but you don't have to.
8. After the study and data analysis the recordings will be destroyed.
9. Feel free to ask any questions, or for any clarifications that you may come up during the discussion.

<u>V. BEGINNING QUESTION</u>

What is your biggest health concern?

VI. QUESTIONS TO GUIDE THE DISCUSSION

<u>Preamble;</u>

We noted that the most patients taking TB medications one in four don't take medications as required. This prompted us to develop a mobile health tool the uses both text and voice messages to support patients take their medications as prescribed. During the past one month, we have tested the mobile health tool by sending you people the text and voice messages. The purpose of this FDG is to try understand your perceptions of this mobile health tool.

1. What are your thoughts about the mHealth tool to support TB medication adherence?
2. How do you feel about the text and voice message reminders that you received?
3. What is your opinion, on the usefulness of the voice and text message reminders in encouraging patients to complete their medication duration? Please explain.
4. Do you think the voice and text message reminders can change how you view or take care of your TB? Please explain.
5. Would you recommend we incorporate voice and text messages in a larger study? Why?
6. Suggest and briefly explain how we can further improve TB medication adherence mHealth tool?
7. What is your opinion concerning the content, frequency, and delivery time of the text and voice messages sent to you?
8. Did you have any problems while receiving the text and voice messages?

We have come to the end of our discussion and once again thank you so much for participating in this study as well as for your wonderful contributions.

-END-
